# Steroids for IgA Nephropathy: A #NephJC Editorial on the TESTING trial

**DOI:** 10.1016/j.xkme.2022.100565

**Published:** 2022-11-01

**Authors:** Anand Chellappan, Rachael Kermond, Tiffany Caza, Jade Teakell, Swapnil Hiremath

**Affiliations:** 1 Department of Nephrology, All India Institute of Medical Sciences (AIIMS), Nagpur, India; 2 Sydney Children’s Hospital Network, Sydney, Australia; 3 Arkana Laboratories, Little Rock, Arkansas; 4 Department of Medicine, McGovern Medical School, University of Texas Health Science Center, Houston, Texas; 5 Department of Medicine, University of Ottawa, Ottawa, Ontario, Canada

***#NephJC is a recurring twitter-based journal club. #NephJC editorials highlight the discussed article and summarize key points from the NephJC TweetChat.***

IgA nephropathy (IgAN) is one of the most common glomerular diseases worldwide and carries a high disease burden with 30%-40% of patients developing kidney failure.^1^ Given its heterogeneous course, a combination of clinical, laboratory, and histopathologic parameters are used to identify patients at risk of poor outcomes.^2^ Multiple trials have explored the use of corticosteroids and other immunosuppressive medications in high-risk IgAN. The Supportive Versus Immunosuppressive Therapy for the Treatment of Progressive IgA Nephropathy (STOP-IgAN) trial in 2015 compared immunosuppressive therapy (with cyclophosphamide, azathioprine, and/or oral prednisolone) added to a background of optimized renin angiotensin system blockade, compared to supportive care alone.^3^^,^^4^ Despite a proteinuria reduction, there was no benefit in reducing kidney failure. The Therapeutic Evaluation of Steroids in IgA Nephropathy Global (TESTING) trial examined the effect of oral methylprednisolone compared to placebo in high-risk patients (with ≥1 g proteinuria).^5^ Though fewer patients on corticosteroids reached the composite endpoint of a 40% decrease in estimated glomerular filtration rate (eGFR) or kidney failure, firm conclusions regarding efficacy could not be made as the trial was halted early, from an increase in serious adverse events including infections and deaths in the steroid arm.

Given the uncertainty around this topic, the Kidney Disease: Improving Global Outcomes (KDIGO) 2021 Glomerular Diseases Work Group suggests consideration of steroid therapy in IgAN with persistent proteinuria only at a 2B level.^6^ The TESTING investigators studied use of a lower dose methylprednisolone to reduce its adverse effects and provide a more conclusive answer in this setting.^7^

## The Trial

This was a randomized, double-blind, placebo-controlled, multicenter trial.^7^ A 12-week run-in period was utilized to optimize background therapy with renin angiotensin system blockade. Those who had persistent proteinuria and satisfied inclusion criteria (biopsy-proven IgAN, proteinuria >1 g/day, and eGFR 20-120 mL/min/1.73 m^2^), despite treatment adherence, were randomized to receive either oral methylprednisolone or a matching placebo. Methylprednisolone dose was initially 0.6-0.8 mg/kg for 2 months followed by a taper of 6-9 months; however, after the interim stoppage discussed above, this was reduced to 0.4 mg/kg/day for 2 months followed by taper over next 6-9 months. In addition to the lower dose of steroids, antibiotic prophylaxis (sulfamethoxazole-trimethoprim) was also added. Participants were followed monthly for 3 months, then 3 monthly until 1 year, and then annually until completion of the study. The primary outcome was a composite of first occurrence of a sustained (>30 days) 40% decrease in eGFR, kidney failure (requiring dialysis or kidney transplant), or death due to kidney disease. The results reported include these participants and the original cohort as well.

A total of 950 eligible patients were screened at 67 sites around the world. Of these, 503 were randomized, 257 to methylprednisolone and 246 to placebo. Baseline characteristics were consistent in both groups, with male predominance, high prevalence of hypertension, and a median age about 36 years. The primary composite outcome occurred less frequently in the methylprednisolone group compared to the placebo group (74 [28.8%] vs. 106 [43.1%]; hazard ratio, 0.53; 95% CI, 0.39-0.72; *P* < 0.001) over a mean of 4.2 years. Similarly, treatment with methylprednisolone significantly reduced the risk of secondary outcomes including total and rate of eGFR reduction and protein excretion. Importantly, serious adverse effects were significantly higher in the methylprednisolone group compared with placebo (37 vs 8 total events that occurred in 28 [10.9%] vs 7 [2.8%] subjects). These serious adverse effects included hospitalization because of serious infection or gastrointestinal bleeding, clinically evident fractures, new onset diabetes, and 4 fatalities in the methylprednisolone group. The reduction in methylprednisolone dose part way through the study and the addition of antibiotic prophylaxis did mitigate but not eradicate these events.

## The TweetChat

The NephJC tweetchats on the TESTING trial had a total of 154 active participants who tweeted 912 times. A Twitter poll was conducted before the tweetchat with the question: “Following TESTING 1.0 do you use glucocorticoids in individuals with high-risk IgAN?” Of the 80 respondents, about 30% were unsure or reluctant, and another 56% would use steroids depending on the patient characteristics.

The strength of the study was felt to be in the large sample size, stringent inclusion criteria, and double-blind randomization. However, it was pointed out that despite being the largest ever trial in IgAN, which is the commonest glomerulonephritis, TESTING still had only around 500 participants. The protocol had undergone several amendments, including changes in the intervention, population, endpoints, and sample size. Despite the hiccups along the path, the investigators of TESTING were determined to pursue the trial to completion, and these amendments were done transparently with a clear rationale, which the chat participants felt was worthy of appreciation. The knowledge gaps existing in the treatment of IgAN, the unclear role of steroids, and the unavailability of targeted and affordable treatment options apart from steroids were discussed at several points during the tweetchat. The participants questioned the exclusion of Henoch-Schonlein purpura (a form of IgA vasculitis) and secondary IgAN from the trial. The inclusion of people with diabetes or obesity, despite their propensity for steroid-related adverse effects, was considered a pragmatic decision. Though the follow-up (median 4.2 years) was considered short by some given the long course of IgAN, the trial did have enough participant-time to accrue sufficient endpoints. The participants of TESTING are also invited to a post-trial observational cohort study (TESTING-ON) to assess the long-term effects of the intervention.

The reboot of TESTING after the pause for the serious adverse effects noted in the earlier TESTING trial came with a reduction in steroid dose and the addition of prophylaxis against Pneumocystis Jirovecii Pneumonia (PJP). The participants were divided in their opinion on the use of PJP prophylaxis in glomerular diseases treated with steroids before these data. However, in consensus, many agreed on the use of PJP prophylaxis in patients on long-term steroids of more than 20 mg prednisone equivalence. Also, the addition of PJP prophylaxis to the low-dose TESTING regimen was considered a wise addition because 4 cases of PJP pneumonia had occurred in the full dose TESTING cohort. On the topic of adverse effects, the TESTING trial did describe several adverse events but was not informative about other common steroid side effects that impact the quality of life, such as weight gain, impaired glucose tolerance, blood pressure fluctuations, insomnia, and mood disturbances.

**Figure 1:**
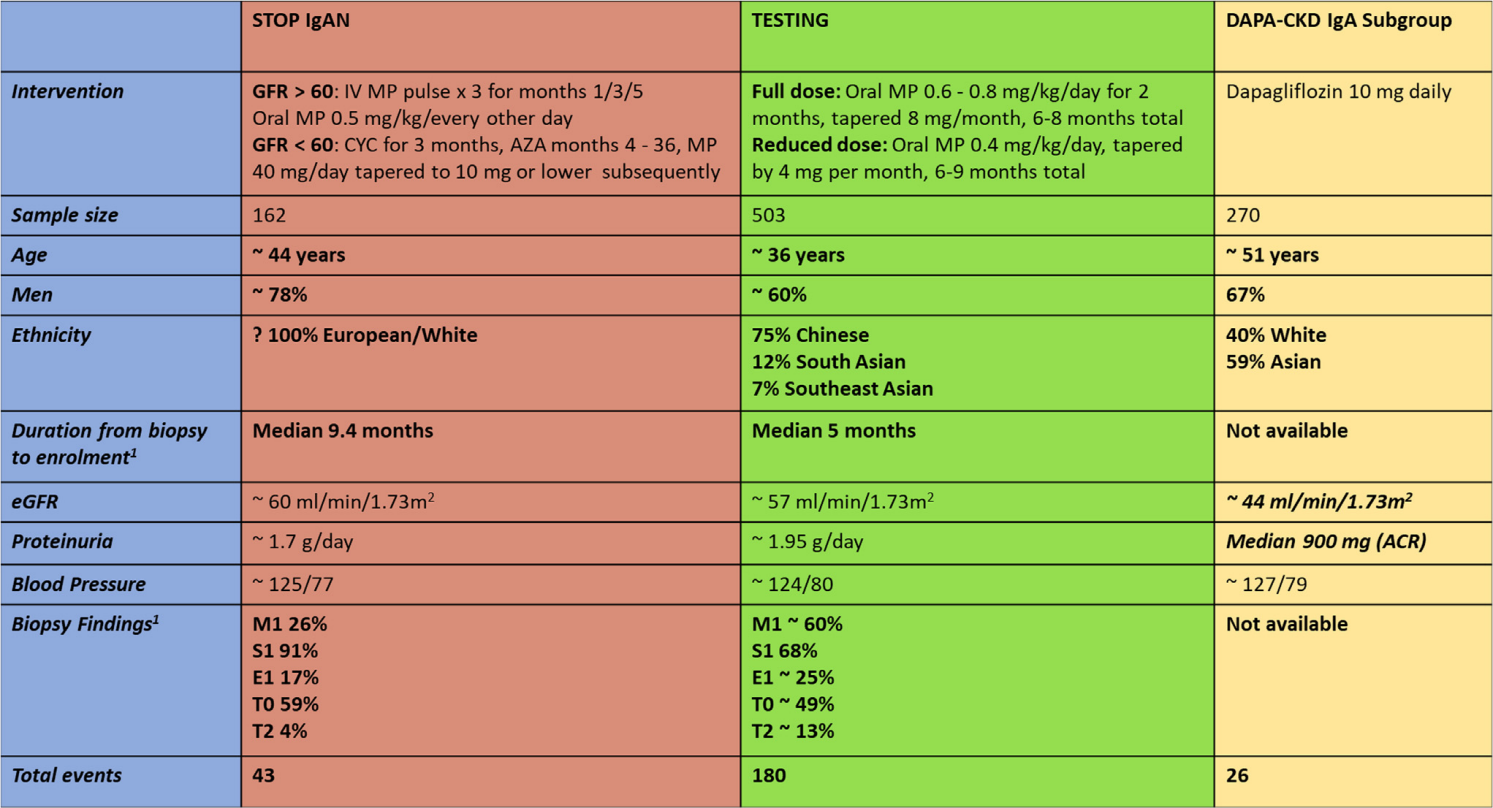
A comparison of baseline characteristics of 3 landmark trials of IgA nephropathy, highlighting differences in the enrolled populations. The population in the TESTING trial was younger, with less time from biopsy and less chronicity on biopsy compared to STOP-IgAN (though the data was from a subgroup of 70 with biopsy data available). The DAPA-CKD population had no biopsy data but was comprised of older individuals with lower GFR, less proteinuria, and lower eGFR, again suggesting an advanced disease compared to TESTING. ^1^ This is a subgroup from the original cohort, of 70 participants from a post hoc analysis. Abbreviations: ACR, albumin-to-creatinine ratio; AZA, azathioprine; CYC, cyclophosphamide; DAPA-CKD, Dapagliflozin and Prevention of Adverse Outcomes in Chronic Kidney Disease; eGFR, estimated glomerular filtration rate; GFR, glomerular filtration rate; IV, intravenous; MP, methylprednisolone; STOP-IgAN, Supportive Versus Immunosuppressive Therapy for the Treatment of Progressive IgA Nephropathy; TESTING, Therapeutic Evaluation of Steroids in IgA Nephropathy Global.

A major limitation of TESTING was considered to be the enrolled population, which was mostly Chinese, thus limiting generalizability. IgAN is known to have geographical variation.^8^ In stark contrast, STOP-IgAN enrolled a White European population and did not show a benefit with immunosuppression. However, STOP-IgAN was different in many other ways also, including a longer time from biopsy, enrolling an older population, and with more chronicity in the biopsy (see Fig 1). Thus, one could consider the patients in STOP-IgAN as being further along the course of disease than those enrolled in TESTING to be a potential explanation of the discordant findings.

**Figure 2:**
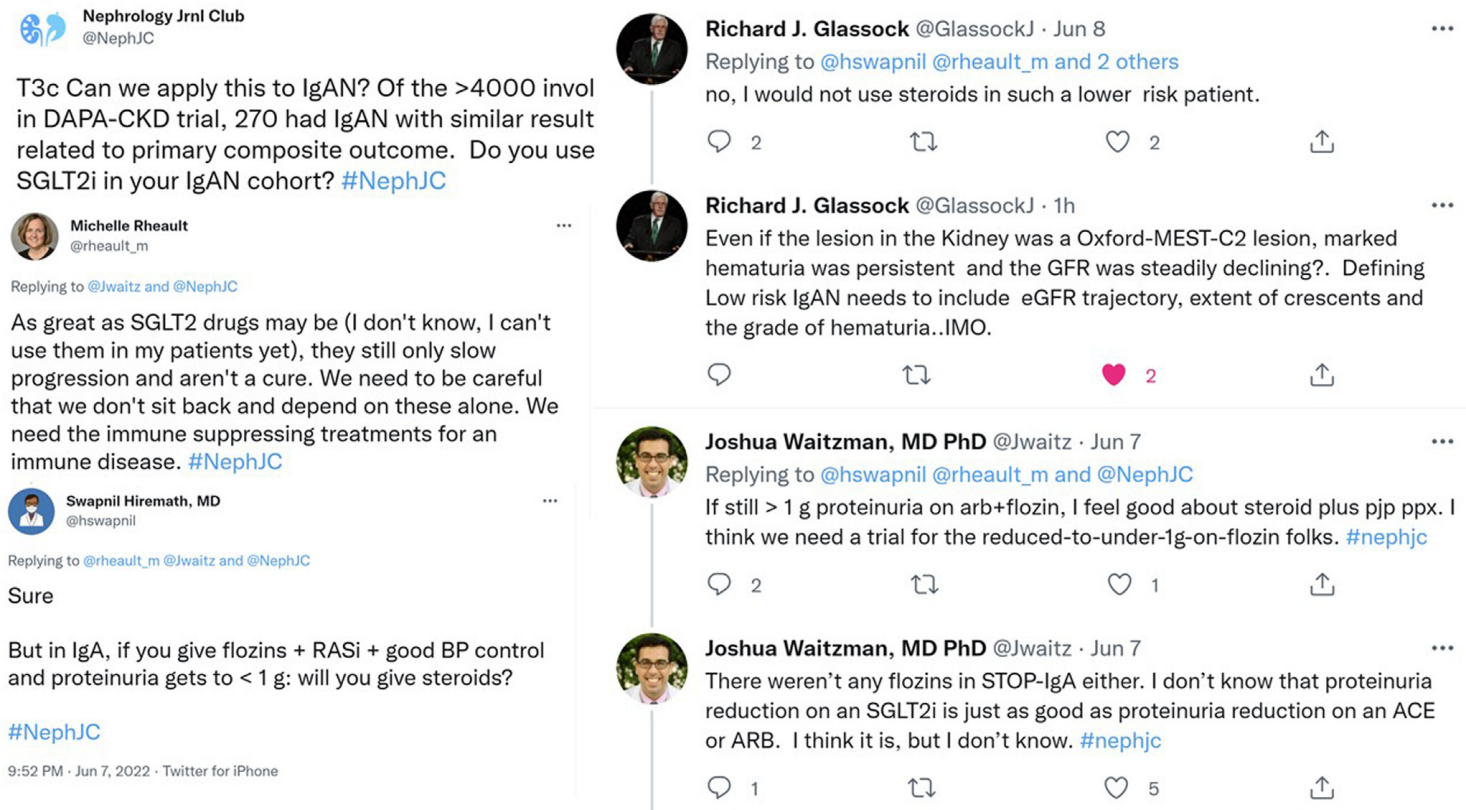
A conversation on the role of sodium/glucose cotransporter 2 (SGLT2) inhibitors or flozins in IgA nephropathy.

The tweetchat discussion got interesting when attention was diverted to the ‘elephant in the room’—the use of sodium/glucose cotransporter 2 (SGLT2) inhibitors, or flozins, in IgAN. A prespecified subgroup analysis of the Dapagliflozin and Prevention of Adverse Outcomes in Chronic Kidney Disease (DAPA-CKD) trial showed that the use of SGLT2 inhibitors in IgAN reduced the risk of chronic kidney disease progression with a favorable safety profile.^9^ Though use of flozins (ie, flozination) at baseline with renin angiotensin system inhibition is now considered an obvious decision, this was not a component of the TESTING protocol given the lack of the data on the benefit of flozins at the time. Secondly, the population in DAPA-CKD was quite different (older, lower eGFR, lower proteinuria) than TESTING, suggesting it was a lower risk and more chronic IgAN (see Fig 1). Lastly, flozins, unlike steroids, would not act on the immunological injury of IgAN (see Fig 2 for a synopsis of the conversation). This again highlighted the need for better-targeted therapies in IgAN, several of which were brought up as being approved or being promising, including endothelin receptor antagonists and targeted release budesonide.

## Conclusion

On the important question of whether steroids are worth the risk, after the tweetchat, nearly 60% of the participants felt that the final results of the TESTING trial would make them reach out for ‘low-dose’ steroids with PJP prophylaxis. Of note, the lower dose of steroids used in TESTING would still mean 35-40 mg prednisone for an average 70 kg individual. It is also important to note that steroids are the only available option in resource-limited settings, and any new drug (eg, targeted release budesonide) for IgAN would be more expensive. There was also interest in more data on the TESTING substudies in the future, which could help clinicians make informed decisions such as the impact of pathology (MEST-C score, comprising of mesangial and endocapillary hypercellularity, segmental sclerosis, interstitial fibrosis/tubular atrophy, and the presence of crescents) on the steroid treatment response, short- and long-term outcomes, and the validation of the IgAN risk prediction model on the TESTING cohort.
